# Efficacy and safety of nanoliposomal irinotecan plus 5-fluorouracil and l-leucovorin in rare histological subtypes of pancreatic cancer

**DOI:** 10.1093/jjco/hyag044

**Published:** 2026-03-20

**Authors:** Tomonao Taira, Tomoyuki Satake, Go Igarashi, Kanae Inoue, Taro Shibuki, Masataka Amisaki, Mitsuhito Sasaki, Hideaki Takahashi, Hiroshi Imaoka, Shuichi Mitsunaga, Masafumi Ikeda

**Affiliations:** Department of Hepatobiliary and Pancreatic Oncology, National Cancer Center Hospital East, 6-5-1, Kashiwanoha, Kashiwa, Chiba 277-8577, Japan; Department of Hepatobiliary and Pancreatic Oncology, National Cancer Center Hospital East, 6-5-1, Kashiwanoha, Kashiwa, Chiba 277-8577, Japan; Department of Hepatobiliary and Pancreatic Oncology, National Cancer Center Hospital East, 6-5-1, Kashiwanoha, Kashiwa, Chiba 277-8577, Japan; Department of Hepatobiliary and Pancreatic Oncology, National Cancer Center Hospital East, 6-5-1, Kashiwanoha, Kashiwa, Chiba 277-8577, Japan; Department of Hepatobiliary and Pancreatic Oncology, National Cancer Center Hospital East, 6-5-1, Kashiwanoha, Kashiwa, Chiba 277-8577, Japan; Department of Hepatobiliary and Pancreatic Oncology, National Cancer Center Hospital East, 6-5-1, Kashiwanoha, Kashiwa, Chiba 277-8577, Japan; Department of Hepatobiliary and Pancreatic Oncology, National Cancer Center Hospital East, 6-5-1, Kashiwanoha, Kashiwa, Chiba 277-8577, Japan; Department of Hepatobiliary and Pancreatic Oncology, National Cancer Center Hospital East, 6-5-1, Kashiwanoha, Kashiwa, Chiba 277-8577, Japan; Department of Hepatobiliary and Pancreatic Oncology, National Cancer Center Hospital East, 6-5-1, Kashiwanoha, Kashiwa, Chiba 277-8577, Japan; Department of Hepatobiliary and Pancreatic Oncology, National Cancer Center Hospital East, 6-5-1, Kashiwanoha, Kashiwa, Chiba 277-8577, Japan; Department of Hepatobiliary and Pancreatic Oncology, National Cancer Center Hospital East, 6-5-1, Kashiwanoha, Kashiwa, Chiba 277-8577, Japan

**Keywords:** pancreatic cancer, second-line therapy, systemic chemotherapy, treatment outcome

## Abstract

**Background:**

Pancreatic cancers other than pancreatic ductal adenocarcinoma (PDAC) are rare and heterogeneous, accounting for fewer than 5%–7% of all pancreatic cancers. These include acinar cell carcinoma, undifferentiated carcinoma, adenosquamous carcinoma, colloid carcinoma, neuroendocrine carcinoma (NEC), mixed neuroendocrine–non-neuroendocrine neoplasm (MiNEN), and invasive intraductal papillary mucinous carcinoma (IPMC). Optimal treatment strategies, including sequencing and later-line options, remain unclear. Although nanoliposomal irinotecan (nal-IRI) plus 5-fluorouracil (5-FU) and l-leucovorin (LV) is effective in gemcitabine-refractory PDAC, its role in these rare subtypes is unknown.

**Methods:**

We retrospectively analyzed nine patients with one of these rare subtypes who received nal-IRI plus 5-FU and LV between June 2020 and November 2024. Efficacy and safety were evaluated.

**Results:**

The cohort included two cases each of IPMC and adenosquamous carcinoma, and one case each of colloid carcinoma, undifferentiated carcinoma, acinar cell carcinoma, NEC, and MiNEN. Partial responses were observed in four patients, including undifferentiated carcinoma, acinar cell carcinoma, NEC, and MiNEN, even among tumors refractory to gemcitabine- or platinum-based regimens. Disease control was achieved in seven patients (77.8%). The median progression-free survival was 6.8 months. Disease control exceeding 12 months was observed in three patients. Median overall survival from first-line therapy was not reached. Treatment-related toxicities were generally manageable, with neutropenia being the most common grade ≥ 3 adverse event.

**Conclusion:**

Nal-IRI plus 5-FU and LV showed antitumor activity and was tolerable among the nine patients analyzed in this study, suggesting it may be a therapeutic option in second- or later-line settings for rare pancreatic cancer.

## Teaser

**Mini-abstract** Nal-IRI plus 5-FU and LV showed antitumor activity and was tolerable among the nine patients analyzed in this study, suggesting it may be a therapeutic option in second- or later-line settings for rare histological subtypes of pancreatic cancer.

## Introduction

Pancreatic ductal adenocarcinoma (PDAC) accounts for more than 90% of all pancreatic malignancies and is characterized by a highly aggressive clinical course and poor prognosis [1, 2]. For patients with unresectable PDAC, standard first-line treatment agents include nanoliposomal irinotecan (nal-IRI) plus 5-fluorouracil (5-FU) and l-leucovorin (LV) plus oxaliplatin (NALIRIFOX) or folinic acid, LV, 5-fluorouracil, irinotecan, and oxaliplatin (FOLFIRINOX), gemcitabine plus nab-paclitaxel (GnP) [3–5]. Based on the results reported from the NAPOLI-1 trial, nal-IRI plus 5-FU and LV became established as an effective regimen in the second-line setting for patients showing disease progression after gemcitabine-based chemotherapy [6, 7].

In contrast, pancreatic cancers other than conventional PDAC, including acinar cell carcinoma, undifferentiated carcinoma, adenosquamous carcinoma, colloid carcinoma, neuroendocrine carcinoma (NEC), mixed neuroendocrine–non-neuroendocrine neoplasm (MiNEN), and invasive intraductal papillary mucinous carcinoma (IPMC), collectively account for fewer than 5%–7% of all pancreatic cancers [8–10]. These rare tumors exhibit diverse histological and molecular characteristics, which precludes straightforward application of PDAC-derived treatment paradigms to patients with these tumors [11]. Due to their rarity, robust clinical evidence to guide optimal management of these patients is still lacking, and treatment strategies, particularly for patients with advanced disease, are generally extrapolated from PDAC treatment data or derived from small case series and institutional experience [12]. Among available regimens, given its proven efficacy in patients with PDAC and mechanistic plausibility, nal-IRI plus 5-FU and LV may represent a rational treatment option for patients with these rare histological subtypes of pancreatic cancer who have previously received systemic therapy, particularly those who show disease progression during treatment with gemcitabine-based regimens. However, reports of clinical studies specifically evaluating this combination in patients with rare pancreatic cancer subtypes are extremely limited.

Given this background, herein, we report a series of nine patients with rare histological subtypes of pancreatic cancer, unresectable at diagnosis, who showed disease progression during previous systemic therapy and were treated with nal-IRI plus 5-FU and LV, focusing on the treatment outcomes and the potential role of this regimen for patients with these rare tumors for which treatment sequencing beyond first-line treatment has not yet been established.

## Materials and methods

### Patients

We retrospectively reviewed the medical records of 275 patients with unresectable or recurrent pancreatic cancer who received nal-IRI plus 5-FU and LV at our institution between June 2020 and November 2024. Among these, patients with rare pancreatic malignancies confirmed by histopathology, excluding pancreatic ductal adenocarcinoma, were identified for inclusion in the present analysis. The rare pancreatic malignancies in the patients included in this study included invasive IPMC, adenosquamous carcinoma, colloid carcinoma, undifferentiated carcinoma, acinar cell carcinoma, NEC, and MiNEN.

### Treatment

Nal-IRI was administered at the dose of 70 mg/m^2^, followed by l-leucovorin at 200 mg/m^2^ and 5-FU at the dose of 2400 mg/m^2^ as a 46- h continuous infusion, repeated every two weeks according to the institutional protocol. Dose modifications were permitted based on the emergence of toxicities or other clinical judgment.

### Clinical evaluation and follow-up

Clinical data of the patients, including the demographic characteristics, treatment details, and clinical outcomes, were extracted from their electronic medical records. Radiological assessment by contrast-enhanced computed tomography (CT) was generally performed at ~8-week intervals, with the exact timing determined by the treating physician in routine clinical practice. The radiological findings were reviewed by experienced radiologists, and the histopathological diagnoses were confirmed by board-certified pathologists. Treatment response was assessed according to Response Evaluation Criteria in Solid Tumors (RECIST) version 1.1, and adverse events were graded using Common Terminology Criteria for Adverse Events (CTCAE) version 5.0. Objective response rate (ORR) was defined as the proportion of patients who achieved complete response (CR) or partial response (PR) as their best overall response. Disease control rate (DCR) was defined as the proportion of patients whose best overall response was CR, PR, or stable disease (SD). Progression-free survival (PFS) was defined as the interval from the initiation of nal-IRI plus 5-FU and LV to radiologic disease progression or death from any cause. Overall survival (OS) was defined as the interval from the start of first-line systemic therapy to death from any cause. Swimmer plots were generated using the R software (version 4.5.1; R Foundation for Statistical Computing, Vienna, Austria).

### Ethics approval and patient consent for participation

This study was conducted with the approval of the Institutional Review Board of the National Cancer Center (approval number: 2020–209) and in accordance with the principles of the Declaration of Helsinki. Approval for review of the hospital records was obtained from the Institutional Review Board of the National Cancer Center. The requirement for written informed consent was waived owing to the retrospective nature of the study, and an opt-out approach was used to provide patients with the opportunity to decline participation.

## Results

A total of nine patients (3.3%) with rare histological subtypes of pancreatic cancers met the inclusion criteria for this study. This patient cohort included two patients with IPMC, two patients with adenosquamous carcinoma, and 1 patient each with colloid carcinoma, undifferentiated carcinoma, acinar cell carcinoma, NEC, and MiNEN. The baseline characteristics of the patients are summarized in Table 1. The median PFS was 6.8 months (95% confidence interval: 0.42—not available), while the median OS was not yet reached. Among the nine patients, the ORR was 44.4% (4 of 9), and the DCR was 77.8% (7 of 9).

**Table 1:** Summary of the patient characteristics at the baseline.

	Overall (n = 9)
Age, years	
Median (range)	71 (56–78)
Gender, n (%)	
Male	6 (66.7)
Female	3 (33.3)
ECOG-PS, n (%)	
0	4 (44.4)
1	5 (55.6)
Disease stage, n (%)	
Metastatic	5 (55.6)
Locally advanced	1 (11.1)
Recurrent	3 (33.3)
Metastatic/Recurrence site, n (%)	
Liver	5 (55.6)
Lymph node	4 (44.4)
Peritoneum	3 (33.3)
Lung	3 (33.3)
Histopathological subtype, n (%)	
Invasive intraductal papillary mucinous carcinoma	2 (22.2)
Adenosquamous carcinoma	2 (22.2)
Colloid adenocarcinoma	1 (11.1)
Undifferentiated carcinoma	1 (11.1)
Acinar cell carcinoma	1 (11.1)
Neuroendocrine carcinoma	1 (11.1)
Mixed neuroendocrine-non-neuroendocrine neoplasm	1 (11.1)
Previous chemotherapy regimens, n (%)	
1	8 (88.9)
≧2	1 (11.1)
1st-line regimen, n (%)	
Gemcitabine + nab-paclitaxel	8 (88.9)
Etoposide + carboplatin	1 (11.1)

**Figure 1:**
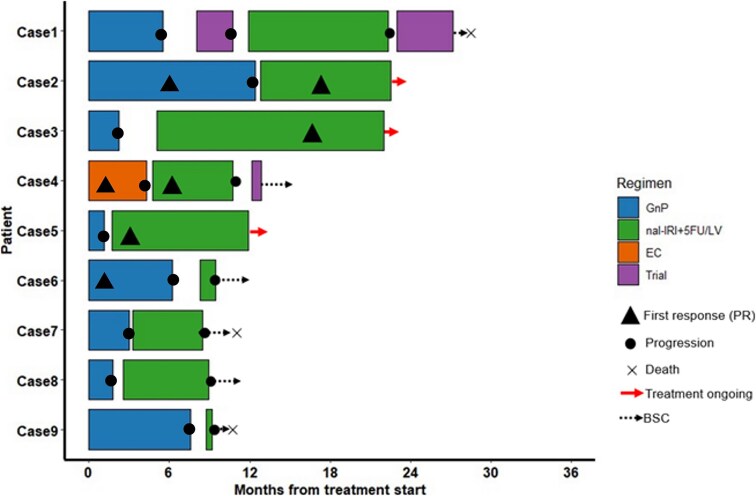
Swimmer plot of the treatment regimens in nine patients with rare non-ductal pancreatic cancers. Each bar represents the treatment duration for an individual patient. Colors indicate different treatment regimens: GnP (blue), nal-IRI + 5-FU/LV (green), EC (orange), and investigational trial regimens (purple). Triangles (▲) denote the timing of the first objective response (PR). Black circles (●) indicate disease progression, and crosses (×) indicate death. Red arrows represent ongoing treatment at the time of data cutoff. Dashed arrows indicate transition to BSC. Data cutoff: August 10, 2025. Abbreviations: GnP, gemcitabine plus nab-paclitaxel; EC, etoposide plus cisplatin; nal-IRI + 5FU/LV, nanoliposomal irinotecan in combination with 5-fluorouracil and l-leucovorin; PR, partial response; BSC, best supportive care.

Detailed summaries of individual cases are presented in Table 2 and Fig. 1. In addition, selected representative cases are described below.

**Table 2:** Individual patient characteristics and treatment outcomes.

Case	Histology	Age	Gender	Disease stage	Prior treatment	nal-IRI plus 5FU and LV Line	Best response	PFS (months)	OS (months)	AE ≧Grade3
1	Colloid carcinoma	71	F	Recurrence	GnP investigational trial	3rd	SD	10.6	28.4	
2	Undifferentiated carcinoma	62	F	Metastatic	GnP	2nd	PR	≥10 (ongoing)	≧23.2 (alive)	Neutropenia, Anemia
3	Acinar cell carcinoma	78	M	Metastatic	GnP	2nd	PR	≥17 (ongoing)	≥22.0 (alive)	Neutropenia, Platelet count decreased
4	Neuroendocrine carcinoma	71	F	Metastatic	EC	2nd	PR	5.9	≥13.1 (alive)	
5	Mixed neuroendocrine-non-neuroendocrine neoplasm	61	M	Metastatic	GnP	2nd	PR	≥10 (ongoing)	≥11.9 (alive)	
6	Adenosquamous carcinoma	74	M	Metastatic	GnP	2nd	PD	2.0	≥11.7 (alive)	
7	Adenosquamous carcinoma	76	M	Locally advanced	GnP	2nd	SD	5.2	11.6	
8	Invasive intraductal papillary mucinous carcinoma	56	M	Recurrence	GnP	2nd	SD	6.8	≥11.3 (alive)	
9	Invasive intraductal papillary mucinous carcinoma	73	M	Recurrence	GnP	2nd	PD	0.8	10.2	Neutropenia

Abbreviations: F, Female; M, Male; nal-IRI + 5FU/LV, nanoliposomal irinotecan in combination with 5-fluorouracil and l-leucovorin; GnP, gemcitabine plus nab-paclitaxel; EC, etoposide plus cisplatin; SD, stable disease; PR, partial response; PD, progressive disease; PFS, progression-free survival; OS, overall survival; AE, adverse event.

### Representative cases

**Figure 2:**
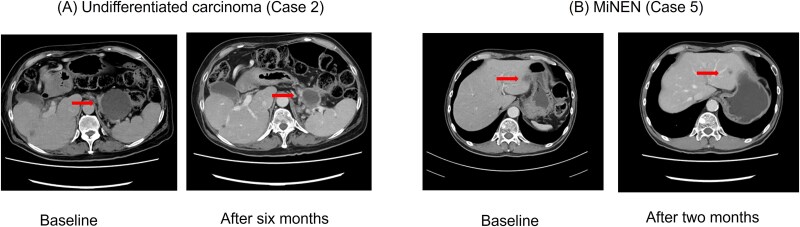
Representative CT images of cases that showed PR. (A) Undifferentiated carcinoma (case 2): Baseline CT obtained before the initiation of treatment with nal-IRI plus 5-FU and LV showed a pancreatic mass (→). Follow-up CT obtained after six months of treatment demonstrated marked tumor shrinkage consistent with partial response. Serum CA19–9 and CEA levels at the baseline were 381 U/mL and 9.3 ng/mL, respectively, the values decreased to 78.7 U/mL and 3.9 ng/mL, respectively, at six months after the start of treatment, when PR was achieved. (B) MiNEN (case 5): Baseline CT obtained before the initiation of treatment with nal-IRI plus 5-FU and LV showed one of multiple hepatic metastatic lesions (→). Follow-up CT obtained after two months of treatment demonstrated marked regression of this representative lesion, consistent with partial response. Serum CA19–9 and CEA levels at the baseline were 329 U/mL and 24.1 ng/mL, respectively; the values changed to 8.2 U/mL and 30.7 ng/mL, respectively, at two months after the start of treatment, when PR was confirmed.

Three representative cases are summarized in the main text, while concise descriptions of the remaining cases are provided in Supplementary Text S1. Case 3 is presented because it demonstrated the longest progression-free survival with nal-IRI plus 5-FU and LV. Cases 2 and 5 are included as clear examples of partial response and are shown together with corresponding radiologic images and tumor marker changes (Fig. 2). Overall, partial responses were observed in four of the nine patients treated with nal-IRI plus 5-FU and LV.

### Undifferentiated carcinoma (Case 2, Fig. 2A)

First-line GnP therapy led to PR by 6 months and was continued until disease progression was detected at 13 months. As the patient maintained an Eastern Cooperative Oncology Group Performance Status (ECOG-PS) score of 1 with preserved organ function and was considered clinically suitable for further systemic therapy, second-line treatment with nal-IRI plus 5-FU and LV was initiated. Grade 3 neutropenia occurred after the first cycle, but the serum tumor marker (Carcinoembryonic Antigen (CEA), Carbohydrate Antigen 19–9 (CA19–9)) levels declined by 1 month, and PR was confirmed at 6 months. Treatment had been continued for 10 months by the time of data cutoff.

### Acinar cell carcinoma (Case 3)

GnP was used as first-line therapy, but disease progression, with the development of new hepatic metastases, was detected after 2 months. As the patient maintained an ECOG-PS of 1 and had adequate organ function, second-line treatment with nal-IRI plus 5-FU and LV was initiated. While the tumor marker (CEA, CA19–9, Alpha-fetoprotein) levels at the baseline were normal, marked decrease of the serum lipase level from 1500 U/L to ~100 U/L, near the normal range, was observed after one month of treatment. PR was achieved at 13 months, and the treatment has been maintained for 17 months with continued response.

### MiNEN (Case 5, Fig. 2B)

First-line GnP was discontinued after 1.5 months due to the onset of delirium, which was not attributed to any organic cause but was suspected to be treatment-related. As the patient maintained an ECOG -PS of 1 with adequate organ function and was considered suitable for further systemic therapy, second-line treatment with nal-IRI plus 5-FU and LV was initiated, during which the neuropsychiatric symptoms did not recur. Serum CA19–9 decreased from ~300 U/mL to within the normal range by 2 months, and the treatment response was assessed as a partial response based on CT findings. The patient remains on treatment, with the response sustained for 10 months.

## Discussion

This study retrospectively analyzed nine patients with rare histological subtypes of pancreatic cancer who received nal-IRI plus 5-FU and LV. To the best of our knowledge, no previous report has comprehensively examined the clinical outcomes of nal-IRI–based therapy across various non-ductal pancreatic cancer subtypes. This study therefore provides new descriptive insight into the potential role of nal-IRI plus 5-FU and LV in patients with rare and heterogeneous subtypes of non-ductal pancreatic cancer. In this patient cohort, nal-IRI plus 5-FU and LV showed promising activity across diverse histological subtypes, including adenosquamous carcinoma, acinar cell carcinoma, NEC, and MiNEN. Disease control was observed across all tumor types, with partial responses observed even in patients whose disease was refractory to first-line therapy. Notably, some patients achieved durable tumor control that was sustained for over 12 months. These findings suggest the therapeutic usefulness of nal-IRI plus 5-FU and LV not only in patients with PDAC, efficacy against which was established in the NAPOLI-1 trial, but also in patients with other rare histological subtypes of pancreatic cancer.

The efficacy of nal-IRI plus 5-FU and LV in PDAC was firmly established by the NAPOLI-1 trial, which demonstrated improved OS as compared with that in gemcitabine-pretreated patients who received 5-FU plus LV [7]. In contrast, evidence for treatments in patients with rare histological subtypes of pancreatic cancer is extremely limited [8]. The only prospective trial identified was the NET-02 phase II study, which evaluated the therapeutic efficacy/safety of nal-IRI plus 5-FU versus docetaxel as second-line therapy in patients with extrapulmonary NEC [13]. Other than the NET-02 study, no published prospective trials have evaluated the use of nal-IRI plus 5-FU and LV in rare histological subtypes of pancreatic cancer. However, several retrospective studies have reported the outcomes of systemic chemotherapy for these rare histological subtypes of pancreatic cancer. For pancreatic acinar cell carcinoma, a multicenter retrospective study reported a median OS of 13.2 months [14]. The median PFS with first- and second-line chemotherapies were 2.7 and 3.9 months, respectively. In the second-line setting, the ORR and DCR were 24% and 56%, suggesting the potential efficacy of platinum- and irinotecan-containing regimens. For pancreatic adenosquamous carcinoma, a multicenter retrospective study reported that first-line GnP and FFX achieved comparable outcomes, with median OS of 7.3 and 7.2 months, median PFS of 2.8 and 2.3 months, and ORRs of 26.9% and 20.0%, respectively [15]. Furthermore, for pancreatic undifferentiated carcinoma a multicenter cohort analysis demonstrated a poor prognosis, with a median OS of 4.08 months. Among first-line regimens, GnP showed a median PFS of 4.6 months and an ORR of 33.3% [16]. Although evidence remains limited, these studies collectively indicate that systemic chemotherapy can provide at least modest clinical benefit across certain rare histological subtypes of pancreatic cancer. Our findings provide complementary evidence to these limited data published previously on the feasibility and potential efficacy of nal-IRI plus 5-FU and LV in rare histological subtypes of pancreatic cancer.

This study had several limitations. First, the present study was a retrospective single-center analysis with a small sample size, which inherently limits the generalizability of its conclusions. Nonetheless, reports describing the use of nal-IRI plus 5-FU and LV in patients with rare histological subtypes of pancreatic cancer are scarce, and this study provides additional descriptive data in this area. Second, although PFS was used as an efficacy indicator, this parameter can be substantially influenced by the intrinsic aggressiveness of each histological subtype. Therefore, caution is warranted when interpreting outcomes derived from a cohort consisting of patients with multiple heterogeneous tumor types. Third, treatment selection and timing were not standardized and were left to the attending clinicians’ discretion, which could have led to potential selection bias. Despite these limitations, the present study provides important preliminary evidence supporting the potential therapeutic role of nal-IRI–based regimens in patients with rare histological subtypes of pancreatic cancer.

In conclusion, nal-IRI plus 5-FU and LV represents a promising therapeutic option for selected patients with rare histological types of pancreatic cancer.

## Supplementary Material

Supplementary_Text_S1_Patient-level_narratives_JJCO_hyag044
