# Supplementary material for: Efficacy and safety of nanoliposomal irinotecan plus 5-fluorouracil and l-leucovorin in rare histological subtypes of pancreatic cancer
Source: Jpn J Clin Oncol. 2026 Mar 20;56(7):858–64. doi: 10.1093/jjco/hyag044 (PMC13334330; doi:10.1093/jjco/hyag044)
Supplement: Supplementary_Text_S1_Patient-level_narratives_JJCO_hyag044 [file supplementary_text_s1_patient-level_narratives_jjco_hyag044.docx]

Supplementary Text S1. Patient-level narratives

Colloid carcinoma (Case 1)

The patient received first-line Gemcitabine plus nab-paclitaxel (GnP) therapy for pulmonary metastases, which resulted in marked tumor regression, including apparent disappearance of the lung lesions. Therefore, the patient underwent conversion surgery. Postoperatively, the patient received adjuvant S-1 therapy for 6 months; however, 6 months after completion of this treatment, pulmonary recurrence occurred. GnP initiated as the first-line therapy for recurrence led to disease progression after 6 months; subsequently, an investigational trial regimen started as second-line therapy failed after 3 months. Third-line therapy with nanoliposomal irinotecan (nal-IRI) plus 5-fluorouracil (5-FU) and leucovorin (LV) was initiated with Eastern Cooperative Oncology Group Performance Status (ECOG-PS) 0 despite the patient having pleural effusion requiring pleurodesis. After two months, both the serum Carcinoembryonic Antigen (CEA) and Carbohydrate Antigen 19-9 (CA19-9) levels improved, and CT showed 26% tumor shrinkage (SD by RECIST). However, one month later (during the third month of treatment), the serum tumor marker levels began to rise again and no further radiological response was observed. Disease control was maintained for 11 months before disease progression was detected. After 5 months of a clinical-trial regimen as fourth-line treatment, the patient was transitioned to best supportive care (BSC).

Neuroendocrine carcinoma (Case 4)

Etoposide plus cisplatin (EC) as first-line therapy resulted in PR at 2 months, but disease progression was detected at 4 months. As the patient maintained an ECOG-PS of 0 and had adequate organ function, second-line treatment with nal-IRI plus 5-FU and LV was initiated. The abdominal pain improved after treatment initiation and the serum tumor marker levels, including CA19-9, CEA, and Neuron-specific enolase, were approximately 50% lower by 2 months, with confirmed PR. The treatment response was maintained for 6 months before disease progression was detected. Third-line trial therapy failed after one month, and the patient was transitioned to BSC.

Adenosquamous carcinoma (Case 6)
First-line GnP therapy resulted in PR at 2 months, however, disease progression was detected at 7 months. Second-line treatment was initiated with nal-IRI plus 5-FU and LV, but tumor bleeding and clinical deterioration occurred at 2 months, prompting transition of the patient to BSC.

Adenosquamous carcinoma (Case 7)

The patient received neoadjuvant chemotherapy with gemcitabine plus S-1 for resectable pancreatic cancer; however, disease progression precluded surgery. Disease progression was detected at 2 months after the start of first-line GnP. As the patient maintained an ECOG-PS of 0 with adequate organ function and was considered suitable for further systemic therapy, second-line treatment with nal-IRI plus 5-FU and LV was initiated.Tumor marker levels were within normal range, making biochemical monitoring uninformative. The first response assessment showed SD, which was maintained for 5 months before disease progression occurred. The patient was subsequently transitioned to BSC.

Invasive intraductal papillary mucinous carcinoma, IPMC (Case 8)
The patient received adjuvant S-1 for 6 months, however, pulmonary recurrence was detected immediately after completion of this treatment. First-line GnP therapy led to disease progression at 2 months. Second-line treatment with nal-IRI plus 5-FU and LV resulted in SD before disease progression was detected at 7 months; the patient was then transitioned to BSC.

Invasive IPMC (Case 9)

After surgical resection followed by 6 months of adjuvant S-1 chemotherapy, the patient developed locoregional recurrence with residual pancreatic and nodal involvement within 6 months of treatment completion. First-line GnP resulted in SD for 8 months before disease progression was detected. At the time of progression, the patient maintained an ECOG performance status of 1 with adequate organ function and was considered suitable for further systemic therapy; therefore, second-line treatment with nal-IRI plus 5-FU and LV was initiated. However, despite the absence of any major adverse events, the patient elected to discontinue therapy after 2 weeks owing to decreased motivation to continue treatment and was subsequently transitioned to BSC.
